# Editorial: Bench to bedside: translating pre-clinical research into clinical trials for childhood brain tumors

**DOI:** 10.3389/fonc.2023.1274465

**Published:** 2023-08-17

**Authors:** Raelene Endersby, Brandon J. Wainwright, Nicholas G. Gottardo

**Affiliations:** ^1^ Brain Tumour Research Program, Telethon Kids Institute, Perth, WA, Australia; ^2^ Centre for Child Health Research, University of Western Australia, Crawley, WA, Australia; ^3^ The University of Queensland Frazer Institute, Translational Research Institute, Woolloongabba, QLD, Australia; ^4^ Department of Paediatric and Adolescent Oncology/Haematology, Perth Children’s Hospital, Nedlands, WA, Australia

**Keywords:** childhood brain tumors, medulloblastoma, ependymoma, high-grade glioma, preclinical, translational, patient-derived xenograft, pediatric

Tumors arising in the brain are the most common solid cancers in children (1). They are the major cause of childhood cancer deaths and worryingly, malignant brain tumor incidence rates have increased 0.5% to 0.7% annually among children and adolescents from 2008 to 2017 (1). Despite improvement in cure rates towards the end of the 20th century, survival statistics have remained unchanged over the past two decades and remain at a level well below that of other childhood cancers, such as leukemia (2), and this remains a major unmet clinical need. Also, survivors have a high risk of significant permanent adverse side effects that require a lifetime of clinical management, significantly impacting health systems and quality of life for patients (3).

The lack of advancements in childhood brain cancer treatment had previously been due to deficiencies in knowledge about the underlying biological causes. However, pediatric neuro-oncology has undergone an exciting and dramatic transformation during the past 20 years, driven by advances in genomic technology, international collaboration, and the generosity of families willing to share tissue samples for research. Next-generation sequencing and other molecular profiling techniques, have revealed that the underlying biology of many childhood brain cancer entities are varied and complex (4–7). Armed with this new knowledge, there is a tremendous opportunity to personalize brain cancer treatment by developing novel therapeutic approaches that are tailored to each molecular subtype of these devastating brain cancers to improve the cure rate while minimizing toxicities.

Most childhood brain tumors are still treated using three main therapeutic modalities: surgery, radiotherapy, and chemotherapy (8). However, there now exists a huge number of new cancer drugs that more precisely target the molecular abnormalities in cancer cells that drive tumor growth or immunotherapies. We now have a major task ahead in identifying the best new drug or therapy for each type of brain cancer and ensuring that clinical trials are applying new treatments in patients with the most likely chance of benefit. Added to this is challenge now is that we have far more new drugs to assess through clinical trials than the number of patients available. We believe the answer to this conundrum is to increase the rigor of preclinical testing to identify only the most effective drugs and prioritize only those drugs with the best chance of success for clinical translation. It is important to note that to date, very few new drugs are yet to demonstrate improved clinical outcomes when translated to early phase trials. There are two major reasons for this. Firstly, there has been a failure to fully evaluate these new drugs in model systems that accurately reflect the disease, or specific molecular subtypes of central nervous system (CNS) cancers, prior to clinical trial. Secondly, not adequately assessing drugs for their ability to penetrate through the brain’s natural protective barrier, the Blood-Brain Barrier (BBB). This means that the drugs fail to reach their target, the tumor.

This Research Topic aims to address these challenges and raises important considerations in preclinical childhood brain cancer research. Importantly, within this Research Topic, Walker provides important perspective, highlighting that a focus on survival as the primary outcome for clinical trials can often fail to recognize the acquisition of brain injury that occurs and persists for the remaining life of brain cancer survivors. From a societal perspective, the economic benefit of reducing cancer therapy-induced late effects is significant, yet preclinical studies often fail to assess or report on long term side effects. While seeking new clinical trials to improve survival, the long term impacts of therapy and quality of life for survivors must also remain a priority. There is an opportunity for the field to better assess the developmental impacts of experimental therapies using preclinical models, and such studies – potentially investigating neurostructural impacts of treatment or behavioral changes – should be valued and encouraged. An example of the balance between brain tumor therapy and quality of life is described by Walker et al. in the setting of optic pathway and hypothalamic glioma (OPHG). Guided by careful analysis of retrospective clinical data, they pose that multi-disciplinary factors should be taken into account when selecting patients for observation versus treatment. Especially given that up to half of OPHG cases are associated with the inherited cancer predisposition syndrome neurofibromatosis type 1 (NF1), treatment decisions should include oncologists, ophthalmologists, endocrinologists, neurodevelopmentalists and geneticists, among other specialties. Such a “total care” approach is broadly adopted in many world class pediatric oncology centers and is the gold standard internationally.

There are many more promising cancer therapies emerging than can be tested clinically for rare cancers like pediatric CNS tumors. As such preclinical modelling is an essential step that can focus the field on agents active in the brain and prevent the evaluation of ineffective or sub-optimal agents in children. For preclinical models to inform clinical response more accurately, it is important that the methodologies applied in the research setting better represent those of the clinic. Preclinical radiotherapy has been hampered in the past by the inability of small animal radiotherapy devices to precisely target tissues of interest, while sparing normal healthy tissue. Moreover, the linear quadratic model (9), which attempts to simplify preclinical experimentation by replacing fractionated dosing with a mathematically estimated, bioequivalent single dose of radiotherapy, is relied on heavily for ease of application. However, such differences in dosing schedule, especially when assessing combinations of small molecules with radiotherapy can potentially lead to misleading results. More recent preclinical radiotherapy equipment, such as SARRP (Xstrahl) and SmART+ (Precision X-ray Irradiation) platforms, enable collimated and more accurate delivery of radiotherapy to small animals, which reduces off-target exposure and facilitates fractionated dosing. Such systems are now considered the gold standard for preclinical radiotherapy research. In this Research Topic, Knox et al. report a fractionated radiotherapy protocol they designed and evaluated in a preclinical model of diffuse midline glioma (DMG). Studies such as these are essential to improve how we evaluate radiosensitizing strategies for childhood brain cancers.

*In vivo* preclinical testing is highly valued as it is better able to mimic drug distribution, metabolism, and excretion compared to *in vitro* testing. Different routes of administration and schedules can be evaluated, with pharmacokinetic studies and pharmacodynamic measures of drug action within tumor cells essential in the interpretation of any effect of a novel compound against CNS tumor cells in an orthotopic setting (or lack thereof). While the use of small animals remains fundamental in cancer research, there is an obligation to consider alternative methods that may reduce the number of animals used in experimentation, to support their welfare and enable researchers to use animal models only when strictly necessary. As mentioned above, the BBB limits the brain penetration of many anti-cancer drugs. However, it is well established that the integrity of the BBB can be compromised in tumors and is often more accurately referred to as the blood-brain-tumor-barrier (BBTB). Several recent studies, summarized in this Research Topic by Morris et al., report that the BBTB has different properties across pediatric brain cancer types. They also discuss innovative and complementary model systems that can be used as an alternative to small mammal research, such as zebrafish or intricate 3-dimentional co-culture models of vasculature and tumor cells. Given experimentation in mice is laborious, costly, and requires specialist research and veterinary skills, the application of diverse model systems in preclinical CNS cancer research may facilitate a more rapid and refined selection of which agents should progress to small mammal experimentation. Such an approach is consistent with the 3 Rs (Replacement, Reduction, and Refinement), and ensures the rational and respectful use of laboratory animals.

Translational research is often described as bench to bedside, however a more comprehensive perspective is to think of cancer research as a cycle from bedside to bench and back around. Here, Lazow et al. describe their investigation of a radionuclide therapy, ^177^Lu-DOTATATE (Lutathera^®^), which binds somatostatin receptors and has demonstrated clinical efficacy in adult neuroendocrine tumors, in a pediatric CNS cohort. They show that the targets of Lutathera, somatostatin type-2A receptors (SST2A), are highly expressed in certain high-risk pediatric CNS tumors and meningiomas. This agent is currently being investigated in a phase I/II trial (NCT05278208), and there is now a preclinical opportunity to begin assessing Lutathera in combination with current standard of care therapies with the goal of providing additional information to guide future clinical trial design. Their observation that SST2A has highest expression in non-SHH medulloblastomas, enables this preclinical assessment to be undertaken in the most appropriate models.

As an example of the value of preclinically assessing novel therapies in combination with standard of care therapies, Sengupta et al. describe the assessment of a telomerase inhibitor in the context of high-risk, *MYC*-amplified Group 3 medulloblastoma. In this study, they show that even with very promising *in vitro* and *in vivo* evidence that telomerase inhibition can slow medulloblastoma growth; when the drug imetelstat was combined with radiotherapy, it appeared to antagonize the anti-tumor effects of radiotherapy, although some tumor growth delay was observed (Sengupta et al.). With early phase clinical trials data suggesting this drug is not well tolerated in children (10), this study highlights the value of using preclinical models to undertake proof of concept studies that confirm certain proteins are good therapeutic targets, even if the right clinical agent/formulation is not yet available.

Numerous high quality preclinical studies have provided compelling and convincing evidence of the efficacy of several new drug combinations in a range of pediatric cancer types (examples include (11–13) among others), and these combinations are now being assessed in early phase clinical trials (such as SJ-ELIOT/NCT04023669, SJ-DAWN/NCT03434262, PNOC022/NCT05009992). However, it is too soon to determine if the preclinical effort and data are useful in predicting clinical efficacy. Indeed, the jury is still out regarding the amount and type of preclinical data needed to accurately inform new clinical trials that are more likely to succeed compared to their predecessors. Tackling pediatric brain cancer requires international effort and collaboration. This is especially critical for conducting clinical trials for very rare brain cancer entities. To facilitate and inform clinical trial decision making, Jones et al. brought together multiple international clinical and preclinical consortia to provide a set of specific assessment criteria with respect to *in vitro* and *in vivo* evidence designed to aid prioritization of ideas. These well-considered guidelines provide a benchmark for preclinical pediatric brain cancer studies and encourage rigorous validation of results in multiple different institutions – especially important given non-reproducibility of preclinical data is recognized as a major concern in cancer research (14). While demonstration of treatment efficacy in multiple preclinical models (such as multiple patient-derived orthotopic xenografts (PDOX), murine allograft models, and/or genetically engineered mouse models) is possible for certain high-risk childhood brain cancer entities, this is a significant limitation for other brain cancers where very few models exist, and/or those that do exist fail to represent the spread of diverse molecular subtypes. This is well described by Whitehouse et al., who report on the challenges of developing PDOX models for ependymoma. Pooling data from three different institutions, they describe the poor engraftment rate of human ependymoma in the brain of immune-deficient mice. Moreover, through molecular analyses, their data suggests that certain molecular features might facilitate PDOX model establishment. Clearly, refinements in preclinical modelling are required, with several groups now suggesting that improved success may be achieved in age-appropriate animals that better replicate the developmental stage of the brain in which these tumors arise (Jones et al.; Whitehouse et al.; 15).

Overall, there is significant reason to be optimistic with more clinical options available for children newly diagnosed with CNS cancers or with relapsed disease. Several clinical trials have recently been established based on rationally selected molecularly-targeted therapies and preclinical evidence of efficacy. An exemplary example is PNOC022, an adaptive platform trial for children and young adults with DMGs including diffuse intrinsic pontine gliomas (DIPGs). This has been facilitated in large part through the generous donation of surgical or autopsy tumor tissue for research, establishment of a large number of preclinical DMG/DIPG models, combined with the international collegiality, collaboration and sharing of these models. The molecular analyses of large numbers of high-grade gliomas (HGGs) have identified key mutations which drive gliomagenesis, with certain molecular features now officially recognized in the latest 2021 WHO classification of CNS tumors (16). Of note, is that histone H3.1/H3.3, ATRX and IDH1/2 mutations are frequent in HGG, but currently the diversity of mutations is not well represented in preclinical HGG models, and additional work is required to expand models that harbor H3.3 G34R/V variants. Voon and Wong provide a comprehensive overview of the mechanisms of action for these mutations in HGG, improving our understanding of how these molecular alterations might be targetable therapeutically.

Importantly, epidemiological and molecular data for pediatric brain cancers has mostly relied on North American or European populations, and while these data hint at racial differences among brain tumors, limited global information is available. Yang et al. provide critical data in this Research Topic regarding the experience of the national health center for children in China highlighting some notable differences compared to other reports. With ongoing international generation and sharing of preclinical models, laboratory techniques and molecular data, alongside careful monitoring of ongoing clinical trial findings, and robust scientific discussion, achieving international consensus on the optimal clinical trials to benefit children affected by brain cancer will be achievable.

## Author contributions

RE: Conceptualization, Writing – original draft, Writing – review & editing. BW: Writing – review & editing. NG: Conceptualization, Writing – review & editing.
